# Transformation from acute promyelocytic leukemia in pregnancy to acute myeloid leukemia with MLL-AF9 fusion gene: A case report and literature review

**DOI:** 10.1097/MD.0000000000036403

**Published:** 2023-12-01

**Authors:** Yang Gao, Na Han, Yu Jiang, Ziyuan Lu

**Affiliations:** a Department of Hematology, Guangdong Provincial Key Laboratory of Major Obstetric Diseases, Guangdong Provincial Clinical Research Center for Obstetrics and Gynecology, The Third Affiliated Hospital of Guangzhou Medical University, Guangzhou, China; b Department of Hematology, General Hospital of PLA Southern Theater Command, Guangzhou, China; c Department of Clinical Medicine, Guangzhou Medical University, Guangzhou, China.

**Keywords:** acute myeloid leukemia, acute promyelocytic leukemia, MLL-AF9, transformation

## Abstract

**Rationale:**

Because there are few evidence-based guidelines and an extremely low incidence rate, managing and treating patients who have transitioned from acute promyelocytic leukemia (APL), which was diagnosed during pregnancy, to acute myeloid leukemia (AML), can be difficult.

**Patient concerns:**

In this case, a 34-year-old pregnant patient was diagnosed with APL in medium-risk group in June 2017. After the all-trans retinoic acid and arsenic trioxide-based full-course treatment, the patients achieved complete remission (CR) and were well-tolerated. After 5 years, the patient complained of fatigue for 3 months.

**Diagnosis:**

Bone marrow examination revealed hypercellularity with approximately 50% immunophenotypic abnormal myeloblasts with MLL-AF9 fusion gene. Based on the AML diagnosis criteria of the World Health Organization, the patient was eventually diagnosed with a rare transformation from APL to AML.

**Interventions:**

The patient was treated with two cycles of induction chemotherapy and an allogeneic hematopoietic stem cell transplantation (allo-HSCT).

**Outcomes:**

Until now, the patient is in continuous remission with no signs of APL and AML.

**Lessions:**

Despite the rarity of APL to AML transformation, it is crucial to track the disease’s progress and administer treatment on time. It remains uncertain whether the risk stratification and clinical outcomes of secondary AML with MLL-AF9 are equivalent to those of de novo AML with MLL-AF9. The management and treatment of these patients should be personalized and require further observation.

## 1. Introduction

Acute promyelocytic leukemia (APL) is a distinct subtype of acute myeloid leukemia (AML) characterized by unique cytogenetic changes, molecular pathogenesis, clinical symptoms and therapeutic regimens. Previously, it was a highly fatal disease due to severe coagulation disorders. The introduction of all-trans retinoic acid (ATRA) and arsenic trioxide (ATO) into therapy has significantly improved the clinical efficacy and outcomes with complete remission (CR) rates exceeding 90% and long-term remission rates exceeding 80%.^[1–3]^ As a result, the chemo-free concept has applied to non-high-risk patients and is now recommended by domestic and international guidelines. Despite these advancements, a small minority of APL patients who achieve CR can undergo transformation to other types of AML.^[4]^ The diagnosis and treatment of this transformation remain challenging, as there are no clear guidelines to follow. In this case report, we presented a unique case of APL transformation to AML, which was diagnosed during pregnancy and has not been reported previously. We also described the management and treatment process and conducted the literature review on the transformation of APL into AML.

### 1.1. Case presentation

**Figure 1.**
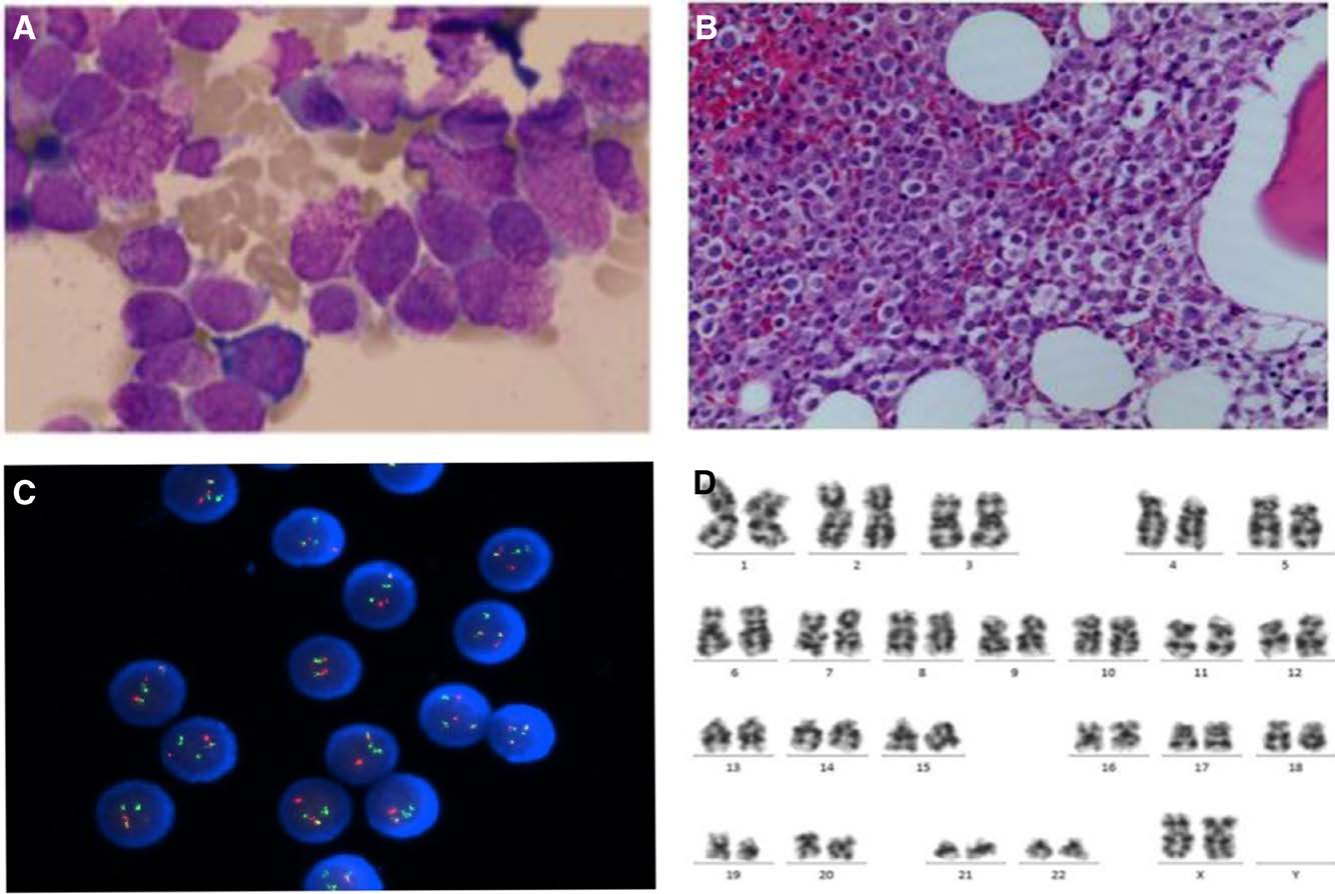
The morphologic and cytogenetic analysis in bone marrow at the time of diagnosis with APL. (A) Morphologic features of bone marrow revealed that hypocellular marrow with a mass of blasts (90%). (Gitter-Giemsa staining, ×1000) (B) Morphologic review of bone marrow biopsy. (Giemsa staining, ×1000) (C) FISH detection of PML-RARα. Green and red fluorescence spots represented PML and RARα location respectively. (DAPI staining, ×1000) (D) Karyotype analysis of bone marrow. APL = acute promyelocytic leukemia, FISH = fluorescence in situ hybridization.

A 34-year-old pregnant patient (G2P1) with no history of hematologic or solid malignancies, and no exposure to toxins or radioactive substances, was diagnosed with APL during the 35th week of gestation on May 27, 2017. She experienced slight vaginal bleeding for one day before her fist presentation, but had no other significant clinical pictures or physical signs. The findings of initial analysis of peripheral blood were as follows: white blood cell count, 0.56 × 10^9^/L; neutrophils count, 0.19 × 10^9^/L; hemoglobin concentration, 90 g/L; platelet count, 19 × 10^9^/L; prothrombin time, 10.8 seconds; activated partial thromboplastin time, 29.5 seconds; fibrinogen, 2.38 g/L; thrombin time, 15.1 seconds; D-Dimer, 2530 ng/mL; creatinine, 37 μmol/L; K+, 3.88 mmol/L; Na+, 137 mmol/L. On May 28, 2017, she underwent a cesarean delivery with red blood cell and platelet infusion, and received ceftriaxone for infection prevention. She delivered a healthy baby. After the cesarean delivery, a bone marrow (BM) aspiration and biopsy were performed on June 2, 2017. The peripheral blood film showed 2% abnormal promyelocytes, while the BM film revealed 85% abnormal promyelocytic blasts with excess of granule and Auer bodied. Additionally, there was dysfunction erythroid differentiation, and rare megakaryocytes and platelets were observed (Fig. 1A). Flow cytometry analysis of the BM identified the presence of abnormal cells (71.9%) that were positive for CD117, CD33, CD13, and CD64, with low expression of CD7, but negative for CD34, HLADR, CD14, CD56, CD4, CD19, and CD20. The BM biopsy results showed extremely active BM hyperplasia (90%) and a mass of blasts (90%), with few erythroid cells and megakaryocytes in BM microenvironment. Immunohistochemical staining of BM biopsy exhibited positive expression of CD117 and CD61, high expression of myeloperoxidase (MPO) (+++), and negative expression of CD235a, CD34, and CD68 (Fig. 1B). Fluorescence in situ hybridization for the PML/RARα fusion gene revealed that 60% of the 200 cells detected in BM showed the presence of the PML/ RARα fusion gene (Fig. 1C). Reverse transcription polymerase chain reaction of PML/RARα also confirmed the presence of the fusion gene in the BM cells. Cytogenetic examination indicated a normal karyotype (Fig. 1D). Based on these findings, the patient was diagnosed with APL (medium-risk group).

### 1.2. Management and treatment

**Figure 2.**
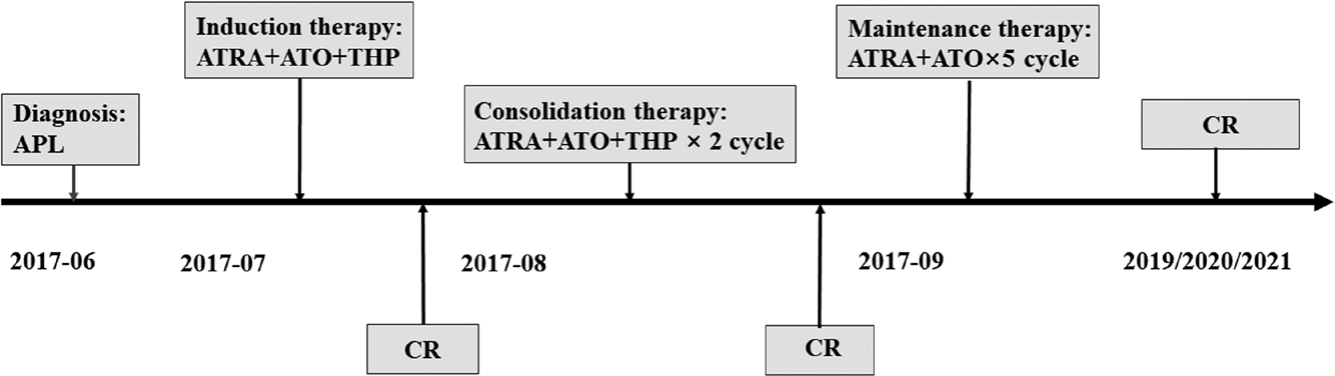
Schedule of treatments and managements in APL period. APL = acute promyelocytic leukemia. ATRA = all-trans retinoic acid, ATO = arsenic trioxide, CR = complete remission, THP = pirarubicin.

According to the APL guidelines of National Comprehensive Cancer Network and Chinese Medical Association, the patient received induction therapy consisting of ATRA, ATO, and pirarubicin (THP). The specific regimen included ATRA at a dose of 20 mg/m^2^/d for 42 days, ATO at a dose of 0.16 mg/kg/d for 42 days, and THP at a dose of 30 mg on days 3 to 6. Following the induction therapy, the patient achieved complete remission (CR) and underwent 2 cycles of consolidation therapy using ATRA, ATO, and THP. Additionally, intrathecal chemotherapy was administered to prevent central nervous system leukemia. On the September 28, 2017, the patient was initiated maintenance treatment with ATO and ATRA for 5 cycles. Throughout the treatment, the patients was tolerant without remarkable complications, and her APL remained CR. Therefore, from 2019 to 2021, the BM examination and PML-RARα fusion gene analyses were performed annually, consistently showing CR of APL (Fig. 2).

**Figure 3.**
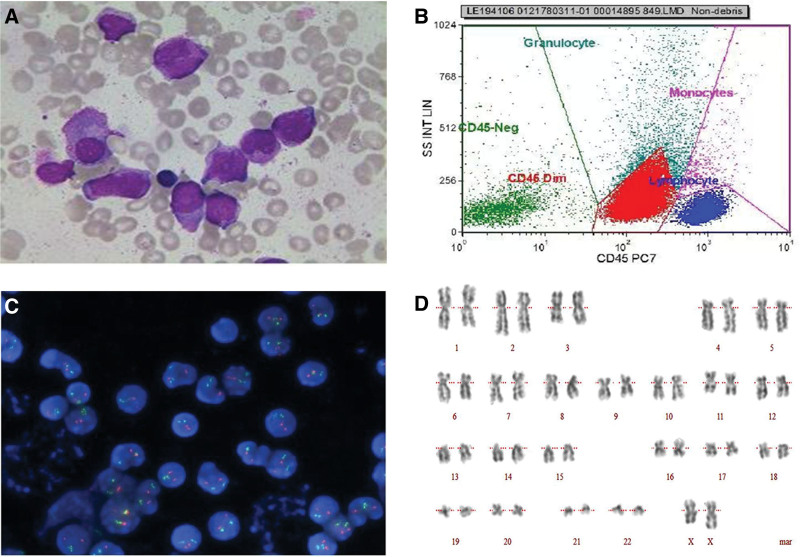
The morphologic, immunologic and cytogenetic results in bone marrow at the time of diagnosis with AML. (A) The bone marrow aspirate showed showing the hypercellularity with 56% myeloblasts. (B) The results of flow cytometry accorded with AML. (C)Detection results of PML-RARα in FISH. (D) Cytogenetic analysis showed a normal karyotype of 46, XX [14]. AML = acute myeloid leukemia, FISH = fluorescence in situ hybridization.

**Figure 4.**
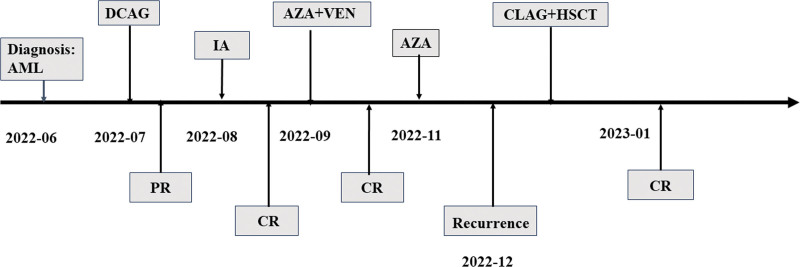
Timeline of interventions and outcomes in AML period. AML = acute myeloid leukemia, DCAG = decitabine + cytarabine + aclacinomycin + G-CSF, PR = partial remission, CR = complete remission, IA = idarubicin + cytarabine, AZA + VEN = azacytidine + venetoclax, CLAG + HSCT = cladribine + cytarabine + G-CSF + allogeneic hematopoietic cell transplantation.

In June, 2022, the patient presented with a complaint of fatigue for three months. The analysis of peripheral blood on June 27, 2022 were as follows: white blood cell count, 2.32 × 109/L; neutrophils count, 0.25 × 109/L; hemoglobin concentration, 85 g/L; platelet count, 23 × 109/L; prothrombin time, 11.7 seconds; activated partial thromboplastin time, 29.9 seconds; fibrinogen, 2.65 g/L; thrombin time, 14.5 seconds; creatinine, 54 μmol/L; K+, 3.66 mmol/L; Na+, 138 mmol/L; Mg2+, 0.91 mmol/L. To establish a diagnosis, a bone marrow aspirate was examined, revealing hypercellularity with 56% myeloblasts. The myeloblasts appeared as moderate-sized cells with round or round-like shapes. The nuclear morphology showed regularity with delicate and flat chromatin, and nucleoli were clearly visible. The cytoplasm exhibited light blue coloration with azurophilic granules, and partial myeloperoxidase (MPO) positivity was observed. Moreover, erythroid cells hyperplasia was inhibited and only one megakaryocyte was observed in the whole film (Fig. 3A). Flow cytometry analysis revealed 53.4% myeloblasts with high expression of CD117, CD123 and CD13, partial expression with CD34, CD33, HLA-DR and CD62, and lack of expression of CD9 (Fig. 3B). The PML-RARα fusion gene were negative based on fluorescence in situ hybridization and quantitative PCR analysis (Fig. 3C). Cytogenetic examination of the BM indicated a normal karyotype of 46, XX [14] (Fig. 3D). The detection of AML-related fusion genes revealed the presence of the MLL-AF9 fusion gene. Next-generation sequencing analysis of gene mutations identified a missense mutation in the DIS3 gene (nucleotide alteration: c.238A > C; amino acid change: p.Ile795Leu; mutation frequency: 48.71%). Taking into consideration the patient’s clinical symptoms and laboratory findings, a diagnosis of AML with MLL-AF9 fusion gene (medium-risk group) was made. Subsequently, the patient received one cycle of decitabine, cytarabine, aclacinomycin and granulocyte colony-stimulating factor (G-CSF) as a combination therapy (decitabine, 20 mg/m^2^, days 1–5; cytarabine, 10 mg/m^2^, every 12 hours, days 1–14; aclacinomycin, 20mg/d, days 1–4; G-CSF, 150 μg/m^2^, q12h, days 0–14) and one cycle of idarubicin and cytarabine (idarubicin, 10 mg/m^2^, days 1–3; cytarabine, 100 mg, every 12 hours, days 1–7). The patient achieved a rapid CR. However, during the consolidation treatment, the patient experienced a sudden recurrence of leukemia after one cycle of azacytidine and venetoclax (azacytidine, 75 mg/m^2^, days 1–7; venetoclax, 100 mg day 1, 20 mmg day 2, 400 mg days 3–28) and one cycle of azacytidine (75mg/m^2^, days 1–7). Subsequently, she underwent cladribine, cytarabine and G-CSF (CLAG) (cladribine, 5 mg/m^2^, d1-5; cytarabine, 2g/m^2^, d1-5; G-CSF 300 mg/d, d0-5) as bridging therapy to prepare for myeloablative allogeneic hematopoietic cell transplantation (allo-HSCT). The conditioning regimens consisted of busulfan (0.8 mg/kg every 6 hours) from days -7 to -3 (total dose 16 mg/kg), etoposide (12 mg/kg) on days -3 and -2 (total dose 24 mg/kg), and cyclophosphamide (60 mg/kg) on days 3 and 4 (total dose 120 mg/kg) (Fig. 4). Peripheral blood stem cells from matched sibling donors were used for transplantation. Following the transplantation, the patient experienced agranulocytosis and infectious complications, which resolved shortly after treatment. On day 40 after allo-HSCT, bone marrow analysis, minimal residual disease analysis, and quantification of the MLL-AF9 fusion gene all showed negative results for leukemia. The blood examination indices were normal again and did not contain primitive cells. Apart from the development of grade I chronic skin graft-versus-host disease, the patient showed no other symptoms and complaints. The patient provided informed consent for publication of this case.

## 2. Discussion

Owing to the advancements in management and treatment of APL, the majority of patients achieve long-term CR and disease-free survival. However, a minority of APL patients still experience refractory/recurrent disease or other complications.^[5]^ One of the most serious complications is the development of secondary neoplasms. Secondary acute myeloid leukemia (sAML) is a term that refers to the development of AML following an antecedent hematologic disorder (secondary AML- antecedent hematologic disorder, sAML-AHD), such as myelodysplastic syndrome, or myeloproliferative neoplasm, or aplastic anemia, or prior exposure to cytotoxic chemotherapy drugs or radiotherapy or immunosuppressive therapy (therapy-AML, t-AML).^[6]^ Across multicenter studies, the incidence of sAML ranged from 10% to 30% of all AML cases. In total, sAML-AHD accounted for 60% to 80% of sAML cases.^[7–9]^ In particular, in APL, the incidence of therapy-related myeloid neoplasms which included myelodysplastic syndrome, AML and acute lymphoblastic leukemia, reported in a few studies for patients in CR ranged from 0.97% to 6.5%. The 6-year cumulative incidence of therapy-related myeloid neoplasms was only 2.2%.^[10]^ Therefore, it is rare for APL to undergo transformation to AML during CR period and the reasons and underlying mechanisms for its occurrence in our case remain unclear.

In a study that evaluated the disease characteristics, treatment response and outcomes of sAML, patients with “sAML” were found to be older, and had poorer WHO performance status and higher rates of adverse karyotypes compared to patients with de novo AML. Similarly, the achievement of CR and survival were significantly inferior in “sAML” patients. Among them, sAML-AHD patients had a higher age and rates of intermediate-risk karyotypes compared to t-AML patients. There were no significant difference between sAML-AHD and t-AML in terms of gender, CR achievement, refractory rates, relapse rates and early death.^[11]^ These findings aligned with data from a Danish national population-based cohort study, which showed that “sAML” patients were older, had lower CR rates, and poorer survival compared to patients with de novo AML. Additionally, patients with sAML-AHD were more likely to have intermediate cytogenetics than patients with t-AML. However, adverse cytogenetics were more frequently found in t-AML patients, which may be a result of the cytotoxic effect of the drugs on hematopoietic progenitors.^[9]^ The drugs associated with t-AML can be mainly divided into 2 classes: alkylating agents (such as melphalan, cyclophosphamide and nitrogen mustard) and topoisomerase-targeted drugs (such as etoposide, doxorubicin, daunorubicin and mitoxantrone). Certian drugs tended to be associated with specific cytogenetic abnormal changes (alkylating agents with 5q and-7 abnormalities and topoisomerase inhibitors with 11q abnormalities). Individual susceptibility to cytotoxic agents also participated in the development of t-AML.^[12]^ These findings provided support for the hypothesis that the development of t-AML may be attributed to the resistance of advantaged clones with specific cytogenetic abnormalities (single hit) to certain cytotoxic drugs, as well as the suppression of cytotoxic treatment in disadvantaged clones (double hit). However, the current studies did not establish a clear relationship between the dose-intensity of cytotoxic treatment and the incidence of t-AML. In the case of sAML-AHD, the transformation may be influenced by the inherent lineage plasticity of leukemia stem cells. In our case, the patient received THP, a topoisomerase inhibitor, during the induction and consolidation therapy. Although the patient was diagnosed with sAML in the cytogenetic intermediate group five years after achieving remission from APL, the potential role of cytotoxic drugs in the development of sAML cannot be excluded.

The mixed lineage leukemia (MLL) gene, located on chromosome 11q23, plays a crucial role in maintaining chromosomal stability. MLL rearrangements can occur with approximately 80 different partner genes.^[13]^ In MLL-rearrangement (MLL-r) AML, the most common fusion partners are AF9 and AF6, resulting in the translocations t (9;11) (q22; q23) and t (6;11) (q27; q23) respectively. MLL rearrangements are often observed in t-AML following treatment with topoisomerase II inhibitors and are associated with poor clinical outcomes.^[14]^ According to current guidelines, except t (4;11) (q21; q23), t (6; 11) (q27; q23), and t (10; 11) (p12; q23) translocations, which are considered adverse-risk cytogenetic abnormalities, all other MLL rearrangements are classified as intermediate risk groups.^[15]^ Several studies have shown that MLL-r patients tend to achieve remission after induction chemotherapy but have high rates of relapse.^[16,17]^ Based on these findings of the above-mentioned studies, the patient in our case received azacytidine and venetoclax as consolidation treatment during the second remission period to prevent disease recurrence. However, the patient experienced early recurrence just four months after remission and had to undergo allo-HSCT. It remains uncertain whether the risk stratification and clinical outcomes of sAML with MLL-AF9 are equivalent to those of de novo AML with MLL-AF9.

In summary, our study confirms the poorer outcomes of sAML following APL compared to de novo AML. Despite the positive outcomes of allo-HSCT in our case, there are certain restrictions, such as insufficient therapeutic observation time. Future developments are anticipated in the areas of basic and clinical research, with a focus on the need for better early diagnosis techniques and treatment regimens for sAML.

## Acknowledgments

We are grateful to all the participants of the study.

## Author contributions

**Conceptualization:** Ziyuan Lu.

**Data curation:** Na Han, Yu Jiang.

**Formal analysis:** Yang Gao.

**Funding acquisition:** Ziyuan Lu.

**Writing – original draft:** Yang Gao, Na Han.

**Writing – review & editing:** Ziyuan Lu.
